# The scope and nature of sexual orientation and gender identity and expression change efforts: a systematic review protocol

**DOI:** 10.1186/s13643-020-01563-8

**Published:** 2021-01-08

**Authors:** David J. Kinitz, Travis Salway, Elisabeth Dromer, Dean Giustini, Florence Ashley, Trevor Goodyear, Olivier Ferlatte, Hannah Kia, Alex Abramovich

**Affiliations:** 1 Division of Social and Behavioural Health Sciences, Dalla Lana School of Public Health, University of Toronto, Toronto, Canada; 2 Faculty of Health Sciences, Simon Fraser University, Blusson Hall 10506; 8888 University Dr., Burnaby, B.C. V5A 1S6 Canada; 3 British Columbia Centre for Disease Control, Vancouver, Canada; 4 Centre for Gender and Sexual Health Equity, Vancouver, Canada; 5 Université de Montréal, Montréal, Canada; 6 Centre de recherche en santé publique, Montreal, Canada; 7 University of British Columbia Biomedical Branch Library, Vancouver, Canada; 8 University of Toronto Faculty of Law, Toronto, Canada; 9 School of Nursing, University of British Columbia, Vancouver, Canada; 10 School of Social Work, The University of British Columbia, Vancouver, Canada; 11 Institute for Mental Health Policy Research, Centre for Addiction and Mental Health, Toronto, Canada

**Keywords:** Conversion therapy, Systematic review, Sexual orientation and gender identity and expression change efforts, Lesbian, gay, bisexual, transgender, and queer (LGBTQ)

## Abstract

**Background:**

Sexual orientation and gender identity and expression change efforts (SOGIECE) are a set of scientifically discredited practices that aim to deny and suppress the sexual orientations, gender identities, and/or gender expressions of sexual and gender minorities (SGM). SOGIECE are associated with significant adverse health and social outcomes. SOGIECE continue to be practiced around the world, despite denouncements from professional bodies and survivors, as well as calls for legislative advocacy to prohibit SOGIECE and protect SGM. There are substantial gaps in the availability of consolidated international research to support and refine legislative proposals related to SOGIECE, including those currently underway to enforce bans in Canada and elsewhere.

We therefore propose the first systematic review of international data on SOGIECE that will outline the scope and nature of these practices worldwide. Specifically, we aim to estimate how many SGM have been exposed to SOGIECE, which sub-groups of SGM experience higher rates of SOGIECE, and how estimates of SOGIECE vary over time and place. In addition, we aim to describe when, where, how, and under what circumstances SGM are exposed to SOGIECE.

**Methods:**

To locate an interdisciplinary swath of papers, nine (9) bibliographic databases will be searched: Medline (OVID), Embase (OVID), PsycInfo and Social Work Abstracts via EBSCO, CINAHL, Web of Science Core Collection, LGBTQ+ Source, and Proquest Dissertations & Theses Global and Sociology Collection (ProQuest). A gold standard search will be developed for Medline and adapted to the other databases. Grey literature will be searched at relevant websites, and reference harvesting will be performed in relevant SOGIECE scientific consensus statements. Two authors will independently screen abstracts/titles, screen full texts, abstract data, and apply risk of bias assessments. A narrative synthesis will be implemented to summarize findings.

**Discussion:**

This review will address the gap in synthesized data regarding the prevalence of SOGIECE, social correlates of SOGIECE, variations of SOGIECE over time and place, and the circumstances, settings, and time-points of SOGIECE exposure. Findings from this review will directly inform ongoing and new legislative efforts to ban SOGIECE and other interventions that aim to stem SOGIECE practices and support SOGIECE survivors.

**Systematic review registration:**

Registration with PROSPERO can be found under the registration number: CRD42020196393.

**Supplementary Information:**

The online version contains supplementary material available at 10.1186/s13643-020-01563-8.

## Introduction

### Background and rationale

#### What are conversion therapy and SOGIECE?

“Conversion therapy,” sometimes referred to as “reparative therapy,” “reintegrative therapy,” or “reorientation therapy,” refers to a set of pseudo-scientific, discredited practices that aim to deny and suppress the sexual orientations, gender identities, and/or gender expressions of sexual and gender minorities^1^ (SGM). Conversion therapy ranges from talk-“therapies” to invasive treatments such as eclectic shock therapies [1]. Given the variability in how conversion therapy is articulated and practiced, a fulsome examination requires a broad definition. To capture the breadth of conversion therapy-related practices, such as a youth speaking to a counsellor who provides advice on repressing sexual attraction, a physician prescribing medication to suppress sex drive, or intentional delay of gender non-affirming care to a transgender (trans) or non-binary person, this project uses the phrase sexual orientation and gender identity and expression change efforts (SOGIECE) [2]. This definition includes, but is not limited to, more formal practices of conversion therapy. SOGIECE settings include religious sites, private and unregulated counsellor’s offices, businesses, and licensed healthcare professional offices, among others [3–5]. Despite the increasing marginalization of professional-conducted SOGIECE in recent years, particularly for gender identity and expression change efforts, many healthcare professionals lack training and support to deliver gender-affirming care and may seek ways to deter their patients from transitioning from the gender aligned with their sex assigned at birth [6–11]. Accordingly, our definition of SOGIECE includes practices that delay transition for trans and non-binary people.

#### The prevalence of SOGIECE:

SOGIECE continue to occur across the globe, including jurisdictions with strong legal protections for SGM, such as Canada [2, 12]. To-date, no attempts have been made to synthesize quantitative prevalence estimates (i.e., using a systematic review methodology). Recent Canadian data estimate that, as of 2019, 20% of sexual minority men have been exposed to SOGIECE and 8% have experienced more circumscribed “conversion therapy” practices [1]. In addition, a 2019 Canadian survey with trans people estimated that 11% have experienced conversion therapy at some time in their lives [13], likely a low estimate given the narrower definition used. In the United States (US), empirical data suggest a lifetime prevalence of SOGIECE exposure of 7–18% among sexual minority (i.e., non-heterosexual) people [4, 5] and 14% among trans, non-binary, and other gender minority (i.e., non-cisgender) people [6]. Approximately half of SGM people exposed to SOGIECE were subjected to these change efforts during childhood or adolescence [4, 5]. Lifetime prevalence of SOGIECE exposure is highest among those born before 2000 [2, 7]; however, at least 3–4% of SGM children and adolescents (born after 2000) are estimated to have been exposed to such practices (likely much higher, owing to the challenges in sampling and surveying youth currently/recently exposed to SOGIECE) [2, 4]. Among US sexual minority populations, up to 60% of those exposed to SOGIECE report experiencing these change efforts in religious settings, while the remainder visited counselors (many unlicensed), psychologists, and psychiatrists [3–5]. Among US gender minority populations, 35% report exposure to SOGIECE in religious settings, with the remainder of SOGIECE occurring in secular settings, including offices of medical doctors and psychologists [7]. Taken as a whole, SOGIECE are highly prevalent and continue to harm SGM worldwide; however, there is a need to more carefully compile and analyze these published estimates to understand how they vary over time, place, social characteristics of participants, and definitions of conversion therapy/SOGIECE.

#### The effects of SOGIECE:

SOGIECE are ineffective, harmful, and often lead to poor psychosocial outcomes. For example, SOGIECE have been associated with poor self-esteem, internalized stigma and discrimination, self-harm, self-hatred, depression, anxiety, and adaptive substance use (i.e., as a form of coping or suppression) [2, 14]. More generally, SOGIECE can lead to isolation from both communities of origin and SGM communities, as many survivors of SOGIECE feel that they have lost years of their lives and are not able to embrace their authentic selves [12, 15]. Most alarmingly, it is estimated that over a third of those who experience SOGIECE attempt suicide [2], a statistic that does not capture those who have died of suicide. More than 40 professional regulating bodies (e.g., American Psychiatric Association, Canadian Psychological Association) and numerous regions (e.g., New York, Malta) have denounced SOGIECE due to its ineffectiveness and detrimental health and social impacts [11, 16–19]. Further, several SGM have spoken about their experiences of SOGIECE through political engagements, media, and books to share how it has impacted their lives (e.g., Muse [20]; Poisson [21]).

There is limited SOGIECE-related research—a critical knowledge gap, given ongoing public policy efforts to end SOGIECE and devise health and social support agendas for those who have experienced these practices. Over the past year, numerous national, regional, and local governments have introduced legislation to ban SOGIECE—with varying degrees of support or opposition across geographic, religious, and political lines. Rigorous research syntheses to support or refine legislative proposals related to SOGIECE are not available at this time. We therefore propose a systematic review of international data on the scope and nature of SOGIECE.

#### Study aim and research question:

The aim of this review is to synthesize quantitative and qualitative literature that addresses the scope and nature of SOGIECE among SGM worldwide. To fulfil this aim, we propose the following research questions:

1. What is the scope of SOGIECE globally? In response to this question, we will estimate how many SGM have been exposed, which sub-groups of SGM experience higher rates of SOGIECE, and how estimates of SOGIECE vary over time and place.
2. What is the nature of SOGIECE globally? In response to this question, we will describe when, where, how, and under what circumstances SGM are exposed to SOGIECE.

#### Definitions:

As there are varying definitions associated with this topic, it is necessary to define how particular terms are being taken up, see Table 1.

**Table 1:** Definitions

Sexual orientation	“a person’s capacity for profound emotional, affectional, and sexual attraction to, and intimate and sexual relations with, individuals of the same gender, of a different gender, or of more than one gender” [11]
Gender identity	“a person’s deeply felt internal and individual experience of gender including the personal sense of the body. Gender identity may be completely male or female or may lie outside the male/female binary” [11]
Gender expression	“a person’s desired external appearance as it relates to social expectations and norms of femininity and masculinity” [11]
Conversion therapy	“any treatment, practice, or sustained effort that aims to repress, discourage, or change a person’s sexual orientation, gender identity, gender modality, gender expression, or any behaviours associated with a gender other than the person’s sex assigned at birth or that aims to alter an intersex trait without adequate justification” [11]
Sexual orientation and gender identity change efforts	SOGIECE are related to conversion therapy in that both sets of practices aim to repress, discourage, or change one’s gender identity, gender expression, and/or sexual orientation; however, SOGIECE additionally include less well defined and advertised practices, which in some cases may not be sustained (e.g., single sessions/conversations) [2, 22, 23]

## Methods

### Protocol and study team

This systematic review protocol follows the PRISMA-P guideline for systematic review protocols and checklist (see Additional file 1 for PRISMA-P checklist) [24]. The systematic review team includes expertise in research methods (DG, TS, OF, AA, HK), substantive areas, including SOGIECE and SGM health (TS, OF, HK, FA, DJK, AA, ED, TG), and biomedical library sciences (DG). The research team will meet regularly throughout the review to identify and resolve challenges, validate and reconcile inclusion/exclusion decisions, and ensure quality and rigour of the review processes. The systematic review protocol has been registered on PROSPERO under the number: CRD42020196393.

### Eligibility criteria

The following criteria will be implemented for screening and selection of studies:

- Language

Language restrictions will be in place for both screening and final inclusion of studies. Literature in French, Spanish, and English will be included due to feasibility and French, Spanish, and English being the languages spoken by members of the research team.

b. Participants

Studies involving SGM, according to definitions provided above, of all ages will be included. SOGIECE practices have been documented across a wide range of ages, countries of origin, genders, gender identities, and sexual orientations. Therefore, no other restrictions will be used with regard to participant populations.

c. Time, geography, and setting

SOGIECE have likely been practiced for decades, if not centuries; as noted above, SOGIECE are practiced across multiple countries and settings. We anticipate that literature on this topic will be sparse; therefore, we will not restrict studies by date, geography, or setting.

d. Study designs

Quantitative, qualitative, or mixed-methods studies will be included in the search, including case studies, case series, surveys, cohorts, interviews, and secondary analyses of existing data. We will screen the citations lists of systematic reviews, commentaries, and letters retrieved from literature searches. We anticipate that quantitative studies will be most relevant to research question 1, regarding the scope of SOGIECE, and qualitative studies will be most relevant to research question 2, regarding the nature of SOGIECE, although these methodological distinctions are not exact. Due to the inclusion of multiple study designs, we will not exclude studies based on sample size or related characteristics. Rather, the limitations of studies will be considered and discussed within the review.

e. Content

All studies that include content related to scope (i.e., prevalence) and/or nature (i.e., descriptions of circumstances, timing, and setting) of SOGIECE will be included.

f. Specific exclusion criteria

Given the inclusion criteria above, studies will be excluded based on the following criteria:

1. Studies about SGM that do not include any reference to SOGIECE
2. Studies that reference SOGIECE in the rationale but do not specifically address our objectives related to the scope and nature of SOGIECE
3. Theoretical or ethical essays on the origins or mutability of sexual orientation, gender identity, or gender expression
4. Ethical essays on the practice of SOGIECE
5. Psychotherapeutic guidelines for SGM-affirming care (except for consensus statements that are components of the grey literature search)

### Information sources

The following indexed medical, health science, nursing, psychology, social work, and social science databases will be searched: Medline (OVID), Embase (OVID), CINAHL, PsycInfo, Social Work Abstracts via EBSCO, Web of Science Core Collection, LGBTQ+ Source, Dissertations & Theses Global (ProQuest), and the Sociology Collection on ProQuest.

In addition to searching the databases above, references of all included full-text articles will be reviewed. Articles identified in this step will also have their references reviewed for inclusion in the study. We will hand-review the reference lists of highly relevant papers (e.g., Turban et al. [7]; Ryan et al. [23]) for additional sources (peer-reviewed and grey literature). Additionally, literature databases of co-authors and affiliated networks will be reviewed and compared to a bibliography of all included articles identified in previous stages to ensure literature saturation.

There will also be a targeted grey literature search focused on the most relevant and robust reference lists of consensus statements issued by health professional organizations about the scientific validity (or lack thereof) of SOGIECE [11].

### Search strategy

Two members of the research team, TS and DG, in consultation with all co-authors, have devised a comprehensive, peer-reviewed search strategy.

Exhaustive searches will be conducted using highly-sensitive strategies given the disseminated nature of the literature. Nine (9) bibliographic databases will be searched: Medline (OVID), Embase (OVID), CINAHL, PsycInfo and Social Work Abstracts via EBSCO, LGBTQ+ Source, Web of Science Core Collection, and Proquest Dissertations & Theses Global and the Sociology Collection (ProQuest). A search will be created in Medline and translated into the requirements of the other databases. In consultation with the principal investigator, the librarian has developed an exhaustive list of concepts and controlled terms based on relevant papers and the expertise of the research team. Searches will be iteratively improved to increase sensitivity by testing optimal combinations of keywords, synonyms, and controlled terms (Table 2). In the absence of controlled terms in the databases for SOGIECE, other related headings will be incorporated. A grey literature search strategy will be created based on a combination of browsing reference harvesting and targeted searching of key websites cited by relevant papers [18, 25–34]. The search strategy will be peer-reviewed using the Peer Review of Electronic Search Strategies (PRESS) checklist (see Additional file 2) [35]. Duplication of papers will be performed in RefWorks before the dataset is loaded into Covidence for title and abstract screening.

**Table 2:** Keywords and controlled terms used in search strategy for a systematic review of sexual orientation and gender identity and expression change efforts

SOGIECE-related concept	SOGIECE keyword search terms (combined using OR Boolean)*	SGM keyword search terms (combined using OR Boolean)*	SGM indexed search terms
Set A: concept of “conversion”	“conversion therap*” “conversion effort*” “conversion practice*”	Bisexual* homosexual* “men who have sex with men” sexual orient* “women who have sex with women” sexual minorit* (gay* or lesbian*) (GLB* or LGB*) queer* two spirit (nonheterosexual* or non-heterosexual*) transgender* “gender divers*” “gender creative” (non-binary AND gender) genderqueer genderfluid “trans wom*” “trans m*” transwom* transm* “gender affirmation” (mtf or ftm) (transfeminine or transmasculine) “sex* reassignment” (gender identity disorder or GID) transsex* transex* gender dysphori*	exp *bisexuality/ exp *homosexuality/ exp *“Sexual and Gender Minorities”/ exp *“Transgender Persons”/
Set B: concept of “repair”	“reparative therap*” “reparative effort *” “reparative practice *”		
Set C: concept of “reorientation”	“reorientation therap*” “sexual reorientation” “gender reorientation” “gender identity reorientation”		
Set D: concept of “change efforts”	“sexual orientation change” “gender identity change” “gender expression change” “psychological attempts to change a person’s gender identity from transgender to cisgender” PACGI		
Set E: others	“ex-gay” “gender acceptance therap*” “reintegrative therap*” “gay cure therap*” “sexual attraction fluidity exploration”		

*Note*: *SOGIECE* sexual orientation and gender identity and expression change efforts, *SGM* sexual and gender minorities

*Two sets to be combined using AND Boolean, to improve specificity

### Study records: data management, selection process, abstraction, items, and bias

To support collaboration and organization among the systematic review team, search results will be uploaded and stored in Covidence—a systematic review software manager. The senior authors will provide training to junior team members regarding the systematic review software and techniques. TS and ED will independently screen titles and abstracts, using Covidence, guided by the above inclusion and exclusion criteria. Disagreements will be resolved by consensus, and when in doubt, articles will be carried forward to full-text review. All titles and abstracts that meet inclusion criteria will then have full texts pulled for review. Full texts will be independently screened by TS and ED, using Covidence. In the event that TS and ED cannot achieve consensus, there will be full discussion, and a third co-author, DJK, will be consulted.

Data collection will utilize a standardized process. A data abstraction tool will be used and include titles, authors, year of publication, and findings relevant to the objectives: nature and scope (Additional file 3). TS and ED will independently abstract data. Calibration activities will be conducted among TS and ED to ensure uniformity in their process. Upon completing data abstraction for 20% of articles, abstractors will meet to discuss and reconcile differences in abstracted information and adjust abstracting procedures going forward, for a list of data items and definitions, see Additional file 3.

We will use an adaptation of the Hoy et al. (2012) risk of bias tool for population-based prevalence studies to evaluate the risk of bias in quantitative studies that are included (see Additional file 4) [36]. This tool has been adapted by a subset of authors (TS, DJK, ED) for applicability to SGM samples. Qualitative articles will be assessed using the CERQual approach that assists in assessing confidence in qualitative findings [37].

### Outcomes and prioritization

The primary outcome of interest is the number of SGM who have *exposure* to SOGIECE, based on the definitions provided above. There is a need to understand the magnitude of SOGIECE worldwide and synthesize prevalence rates to illustrate that this phenomenon requires global attention.

Secondary outcomes include the following:

1. Where—i.e., the setting where SOGIECE occurred. This is relevant to identify levels and forms of policy and legislation that can have bearing on SOGIECE prevention or enforcement of bans.
2. When—i.e., the age and calendar year when SGM are exposed to SOGIECE. This is relevant to inform policy regarding minor and adult protections. Age will be considered based on numerical presentation or categorically using such terms as “minor,” “youth,” and “adult.”
3. Under what circumstances—i.e., reasons and motivations for attending SOGIECE, whether forced or compelled to attend or attending voluntarily.
4. How—i.e., the types of activities constituting SOGIECE.

These primary and secondary outcomes will be presented in summary results tables and narrative form, as appropriate.

### Synthesis of results

Results of the systematic review will be presented in a final report that will be structured according the specific objectives identified above, corresponding to the scope and nature of SOGIECE, and the type of data charted.

Quantitative data will be synthesized to the extent possible given the likely heterogeneity of studies selected. A meta-analysis is likely not possible due to the variability of populations (e.g., differing age groups, gender identities, gender modalities) and definitions of SOGIECE used [2, 7, 23]. Results will therefore be presented using a narrative synthesis with tables [38]. Specifically, tables will be used to display prevalence rates among different subpopulations of SGM, along with other characteristics such as geographic area, population demographics (e.g., age, religious background), and year of study/article production. Analysis of social location and equity factors, such as socioeconomic status, race/ethnicity, gender, and sexual orientation, will be conducted to better understand the nuances of SOGIECE and its impacts across and within various SGM subpopulations. The identification of potential disparities within this area will help to inform equity-oriented and population-tailored responses to SOGIECE, including supports for those who have experienced SOGIECE. Qualitative data will be appraised and combined in the narrative presentation as these data relate to the relevant section—scope and nature. In addition, results will be highlighted and discussed narratively per their relevance and potential to inform policy.

In the final section of the synthesis, we will discuss the limitations of the current literature as per the findings of the review as well as the limitations of the current study. Given the dearth of literature discussing SOGIECE, it is likely that there will be several challenges in clearly identifying robust reports that can independently answer our research questions. Reports are likely to omit various populations and demographics impacted by SOGIECE and to inadequately present information related to the outcomes under study.

## Discussion

This proposed systematic review of the prevalence and scope of SOGIECE will be the first of its kind conducted to date. Two prior systematic reviews have been published on the topic of conversion therapy, to the knowledge of our co-author team [39, 40]. One of these reviews was focused solely on gender minorities and used a relatively limited set of search terms [39, 41]. The other review was solely focused on sexual minorities and did not examine estimates of prevalence [40]. We believe that it is beneficial to simultaneously review literature on SOGIECE targeting sexual orientation, gender identity, and gender expression, given overlap between SGM populations (i.e., some sexual minorities are trans; some gender minorities are queer, bisexual, lesbian, gay), the unspecific nature of some SOGIECE (i.e., some practitioners conflate sexual orientation and gender identity, or primarily target non-conforming gender expressions), and the potential to attend SOGIECE with a practitioner who targets more than one of: sexual orientation, gender identity, and gender expression.

Results from this review will provide the prevalence of SOGIECE across international jurisdictions and summarize associations between social characteristics (gender, gender identity, sexual orientation, age, race, disability, socioeconomic position, religiosity) and SOGIECE exposure. Ecologic factors, such as time, place, and study methods, are expected to modify estimates of SOGIECE prevalence. Particularly useful to inform preventative strategies to stop the harm associated with SOGIECE, this study aims to identify the ages at which SGM are first exposed to these practices, in what settings they take place, and the precipitants of an individual experiencing SOGIECE.

### Dissemination

The impetus for this systematic review is the need for policy makers and legislators to have readily available, scientifically robust, and synthesized evidence to inform policy changes involving SOGIECE. Findings from the proposed systematic review will be beneficial to legislators in Canada and other countries and jurisdictions considering SOGIECE bans, such as Australia and Ireland [42–44]. Furthermore, identifying the scope and nature of SOGIECE will assist health care providers, SGM community leaders and advocates, and SGM people themselves when determining supports needed for those who have experienced these practices. This systematic review will be published in an open-access, international journal that will provide evidence for countries considering or implementing federal, provincial, or municipal bans on SOGIECE. In addition, our findings will be shared with Canadian policy leaders and inform a community-based strategic planning meeting involving survivors, researchers, service providers, and politicians. Lastly, findings will be presented at relevant national and international conferences.

## Supplementary Information

**Additional file 1.** PRISMA-P Checklist

**Additional file 2.** Medline search strategy was peer-reviewed using the Peer Review of Electronic Search Strategies (PRESS) checklist

**Additional file 3.** Sample Medline search strategy – PRESS validated.

**Additional file 4.** Risk of bias tool

## Data Availability

All data and materials included in the study are available from the cited primary data sources.

## Footnotes

1 Sexual and gender minorities refers to lesbian, gay, bisexual, transgender, non-binary, queer, and Two-Spirit (LGBTQ2) people.

## References

[1] Conversion Therapy and SOGIECE. 2020; Available at: https://www.cbrc.net/conversion_therapy_sogiece. Accessed 23 May 2020.

[2] Salway T, Ferlatte O, Gesink D, Lachowsky NJ. Prevalence of exposure to sexual orientation change efforts and associated sociodemographic characteristics and psychosocial health outcomes among Canadian sexual minority men. Can J Psychiatry. 2020. 10.1177/0706743720902629. 10.1177/0706743720902629 PMC7298582 31984758

[3] Flentje A, Heck NC, Cochran BN (2014). Experiences of ex-ex-gay individuals in sexual reorientation therapy: reasons for seeking treatment, perceived helpfulness and harmfulness of treatment, and post-treatment identification. J Homosex.

[4] Mallory C, Brown TN, Conron KJ (2018). Conversion therapy and LGBT youth.

[5] Meanley SP, Stall RD, Dakwar O, Egan JE, Friedman MR, Haberlen SA (2019). Characterizing experiences of conversion therapy among middle-aged and older men who have sex with men from the Multicenter AIDS Cohort Study (MACS). Sex Res Soc Policy.

[6] Turban JL, Beckwith N, Reisner SL, Keuroghlian AS (2019). Association between recalled exposure to gender identity conversion efforts and psychological distress and suicide attempts among transgender adults. JAMA Psychiatry.

[7] Turban JL, King D, Reisner SL, Keuroghlian AS (2019). Psychological attempts to change a person’s gender identity from transgender to cisgender: estimated prevalence across US States, 2015. Am J Public Health.

[8] Wylie K, Knudson G, Khan SI, Bonierbale M, Watanyusakul S, Baral S (2016). Serving transgender people: clinical care considerations and service delivery models in transgender health. Lancet.

[9] Lam JSH, Abramovich A (2019). Transgender-inclusive care. CMAJ.

[10] Scheim AI, Zong X, Giblon R, Bauer GR (2017). Disparities in access to family physicians among transgender people in Ontario, Canada. Int J Transgenderism.

[11] Ashley F (2019). Model law–prohibiting reparative practices.

[12] Venn-Brown A. Sexual orientation change efforts within religious contexts: a personal account of the battle to heal homosexuals. Sensoria A Journal of Mind Brain and Culture. 2015;11(1). 10.7790/sa.v11i1.417.

[13] The Trans PULSE Canada Team (2019). QuickStat #1 – conversion therapy.

[14] Pan American Health Organization, Regional Office of World Health Organization (2012). “Cures” for an illness that does not exist: purported therapies aimed at changing sexual orientation lack medical justification and are ethically unacceptable.

[15] Williams C (2017). #DiscoSexology Part V: an interview with Zucker’s patient.

[16] Butterworth B. Malta just became the first country in Europe to ban ‘gay cure’ therapy. London: Pink News; 2016.

[17] Byne W. No title. Regulations restrict practice of conversion therapy 2016. 10.1089/lgbt.2016.0015 26990275

[18] Canadian Psychological Association. No title. CPA policy statement on conversion/reparative therapy for sexual orientation 2015.

[19] Scasta D, Bialer P, American Psychiatric Association (2013). Position statement on issues related to homosexuality.

[20] Muse E (2015). Affirming sexual orientation and gender identity act, 2015.

[21] Poisson J. “Conversion therapy” survivor shares his story. CBC Radio, Toronto, ON, Canada. 2019. https://www.cbc.ca/radio/frontburner/conversion-therapy-survivor-shares-his-story-1.5207493.

[22] Salway T (2020). Ending conversion therapy in Canada: survivors, community leaders, researchers, and allies address the current and future states of sexual orientation and gender identity and expression change efforts.

[23] Ryan C, Toomey R, Diaz R, Russell S. Parent-initiated sexual orientation change efforts with LGBT adolescents: implications for young adult mental health and adjustment. J Homosex. 2020;67(2). 10.1080/00918369.2018.1538407 PMC10371222 30403564

[24] Moher D, Shamseer L, Clarke M, Ghersi D, Liberati A, Petticrew M (2015). Preferred Reporting Items for Systematic Review and Meta-Analysis Protocols (PRISMA-P) 2015 statement. Syst Rev.

[25] Murchison G, Adkins D, Conard LA, Ehrensaft D, Elliott T, Hawkins LA. Supporting and caring for transgender children. Human Rights Campaign. 2016;11. https://www.hrc.org/resources/supporting-caring-for-transgender-children.

[26] National Centre for Lesbian Rights (2014). Conversion therapy mental health advocate letter.

[27] American Medical Association. LGBTQ change efforts (so-called “conversion therapy”). Chicago: American Medical Association; 2019. p. 1–5.

[28] American Psychological Association (2011). Resolution on appropriate affirmative responses to sexual orientation distress and change efforts.

[29] Canadian Association for Social Work Education (CASWE-ACFTS), Canadian Association of Social Workers (CASW) (2015). Joint statement on the affirmation of gender diverse children and youth.

[30] Canadian Professional Association for Transgender Health. Submission to the Standing Committee on Justice Policy Re: Bill 77, Affirming Sexual Orientation and Gender Identity Act, 2015. 2015.

[31] National Association of Social Workers National Committee on Lesbian, Gay, Bisexual, and Transgender Issues (2015). Sexual orientation change efforts (SOCE) and conversion therapy with lesbians, gay men, bisexuals, and transgender persons.

[32] Gaylesta. U.S. Joint statement on conversion therapy (draft 2.1--10/18/17). 2017; Available at: https://gaylesta.org/USJS-Draft. Accessed 23 May 2020.

[33] Telfer MM, Tollit MA, Pace CC, Pang KC (2018). Australian standards of care and treatment guidelines for transgender and gender diverse children and adolescents. Med J Aust.

[34] Substance Abuse and Mental Health Services Administration. Ending conversion therapy: supporting and affirming LGBTQ youth. HHS Publication No. (SMA) 15-4928 2015.

[35] McGowan J, Sampson M, Salzwedel DM, Cogo E, Foerster V, Lefebvre C (2016). PRESS peer review of electronic search strategies: 2015 guideline statement. J Clin Epidemiol.

[36] Hoy D, Brooks P, Woolf A, Blyth F, March L, Bain C (2012). Assessing risk of bias in prevalence studies: modification of an existing tool and evidence of interrater agreement. J Clin Epidemiol.

[37] Lewin S, Glenton C, Munthe-Kaas H, Carlsen B, Colvin CJ, Gülmezoglu M (2015). Using qualitative evidence in decision making for health and social interventions: an approach to assess confidence in findings from qualitative evidence syntheses (GRADE-CERQual). PLoS Med.

[38] Aromataris E, Munn Z. Joanna Briggs Institute reviewer’s manual, vol. 299. Adelaide: The Joanna Briggs Institute; 2017.

[39] Wright T, Candy B, King M. Conversion therapies and access to transition-related healthcare in transgender people: a narrative systematic review. BMJ Open. 2018;8:e022425. 10.1136/bmjopen-2018-022425 PMC6318517 30580262

[40] Serovich J, Craft S, Toviessi P, Gangamma R, McDowell T, Grafsky E (2008). A systematic review of the research base on sexual reorientation therapies. J Marital Fam Ther.

[41] Ashley F (2019). Trans conversion therapy doesn’t call itself conversion therapy.

[42] Halpin H. A deceptive practice”: Bill to ban LGBTQ conversion therapies passes second stage of Seanad. TheJournal.ie.

[43] Karp P. Gay conversion therapy ban found to be LGBTIQ Australians’ top priority. London: The Guardian; 2018.

[44] Johnson S. Liberal justice minister ‘committed’ to criminalizing conversion therapy, says Edmonton MP. Toronto: Global News; 2019.

