# Supplementary material for: The scope and nature of sexual orientation and gender identity and expression change efforts: a systematic review protocol
Source: Syst Rev. 2021 Jan 8;10:14. doi: 10.1186/s13643-020-01563-8 (PMC7796537; doi:10.1186/s13643-020-01563-8)
Supplement: Supplementary file 3 — Additional file 3. Sample Medline search strategy – PRESS validated. [file 13643_2020_1563_MOESM3_ESM.docx]

**Appendix C. Data abstraction form**

Abstractor:

Author:

Journal:

Date of Publication:

Date of Extraction:

| **Data element** | **Options** | **Explanatory notes** |
| --- | --- | --- |
| Study design | Qualitative  Quant: cross-sectional  Quant: longitudinal  Case report  Case series  Secondary data (qual)  Secondary data (quant)  Other | We anticipate quantitative studies to be most relevant to RQ1 (regarding scope/prevalence of SOGIECE). Qual, quant, and case reports/series will be relevant to RQ2 (“nature” of SOGIECE: when/where/why). |
| Study setting | Clinic; description:  School; description:  Church or religious setting; description:  SGM community sample; description:  Other community sample; description: | Study setting will have a bearing on the kinds of SOGIECE (including SOGIECE settings) that are described. |
| Country, region | Open text | Will categorize post-hoc, depending on number of studies per country/region. |
| Description of sample | Open text | E.g., “trans youth”, “people living with HIV”, “patients of a psychiatric clinic” |
| Sample size (N) | Numeric |  |
| Socio-demographic details (%s or means/medians, range/IQR) | Gender identities  Gender modalities (trans, cis)  Race/ethnicity  Age  Religion  Geographies of residence  Markers of socioeconomic status (income, education, occupation)  Other | Related to RQ1, specifically to identify social correlates of SOGIECE exposure. |
| Prevalence of SOGIECE exposure | Numeric | Numerator, denominator, proportion, and measures of variance (standard error, confidence interval) |
| Prevalence of SOGIECE exposure by subgroups | Numeric, by following, as available:  Gender identity  Gender modality  Age groups  Race/ethnicity sub-groups | Numerator, denominator, proportion, and measures of variance (standard error, confidence interval) |
| Other Notes |  |  |
