# Supplementary material for: The scope and nature of sexual orientation and gender identity and expression change efforts: a systematic review protocol
Source: Syst Rev. 2021 Jan 8;10:14. doi: 10.1186/s13643-020-01563-8 (PMC7796537; doi:10.1186/s13643-020-01563-8)
Supplement: Supplementary file 4 — Additional file 4. Risk of bias tool [file 13643_2020_1563_MOESM4_ESM.docx]

**Appendix D. Risk of bias tool**

Adapted from Hoy D, Brooks P, Woolf A, Blyth F, March L, Bain C, et al. Assessing risk of bias in prevalence studies: modification of an existing tool and evidence of interrater agreement. Journal of Clinical Epidemiology. 2012;65(9):934-9.

| **Item*** | **Criteria** | **Notes (from Hoy)** |
| --- | --- | --- |
| 1. Was the study’s target population a close representation of the national population in relation to relevant variables, e.g. age, sex, occupation? | Yes (LOW RISK): The study’s target population was a close representation of the national population.  No (HIGH RISK): The study’s target population was clearly NOT representative of the national population. | The target population refers to the group of people or entities to which the results of the study will be generalised. Examples: The study was a national health survey of people 15 years and over and the sample was drawn from a list that included all individuals in the population aged 15 years and over. The answer is: Yes (LOW RISK).  The study was conducted in one province only, and it is not clear if this was representative of the national population. The answer is: No (HIGH RISK).  The study was undertaken in one village only and it is clear this was not representative of the national population. The answer is: No (HIGH RISK). |
| 4. Was the likelihood of non-response bias minimal? | Yes (LOW RISK): The response rate for the study was >/=75%, OR, an analysis was performed that showed no significant difference in relevant demographic characteristics between responders and nonresponders  No (HIGH RISK): The response rate was <75%, and if any analysis comparing responders and non-responders was done, it showed a significant difference in relevant demographic characteristics between responders and non-responders. | Examples:  The response rate was 68%; however, the researchers did an analysis and found no significant difference between responders and non-responders in terms of age, sex, occupation and socioeconomic status. The answer is: Yes (LOW RISK).  The response rate was 65% and the researchers did NOT carry out an analysis to compare relevant demographic characteristics between responders and non-responders. The answer is: No (HIGH  RISK).  The response rate was 69% and the researchers did an analysis and found a significant difference in age, sex and socio-economic status between responders and non-responders. The answer is: No (HIGH RISK). |
| 5. Were data collected directly from the subjects (as opposed to a proxy)? | Yes (LOW RISK): All data were collected directly from the subjects.  No (HIGH RISK): In some instances, data were collected from a proxy. | A proxy is a representative of the subject. Examples:  All eligible subjects in the household were interviewed separately. The answer is: Yes (LOW RISK).  A representative of the household was interviewed and questioned about the presence of low back pain in each household member. The answer is: No (HIGH RISK). |
| 6. Was an acceptable case definition used in the study? | Yes (LOW RISK): An acceptable case definition was used.  No (HIGH RISK): An acceptable case definition was NOT used. | For a study on low back pain, the following case definition was used: “Low back pain is defined as activity-limiting pain lasting more than one day in the area on the posterior aspect of the body from the bottom of the 12th rib to the lower gluteal folds.” The answer is: Yes (LOW RISK).  For a study on back pain, there was no description of the specific anatomical location “back” referred to. The answer is: No (HIGH RISK).  For a study on osteoarthritis, the following case definition was used: “Symptomatic osteoarthritis of the hip or knee, radiologically confirmed as Kellgren-Lawrence grade 2-4”. The answer is: LOW RISK. |
| 8. Was the same mode of data collection used for all subjects? | Yes (LOW RISK): The same mode of data collection was used for all subjects.  No (HIGH RISK): The same mode of data collection was NOT used for all subjects. | The mode of data collection is the method used for collecting information from the subjects. The most common modes are face-to-face interviews, telephone interviews and self-administered questionnaires. Examples:  All eligible subjects had a face-to-face interview. The answer is: Yes (LOW RISK).  Some subjects were interviewed over the telephone and some filled in postal questionnaires. The answer is: No (HIGH RISK). |
| 9. Was the length of the shortest prevalence period for the parameter of interest appropriate? | Yes (LOW RISK): The shortest prevalence period for the parameter of interest was appropriate (e.g. point prevalence, one-week prevalence, one-year prevalence).  No (HIGH RISK): The shortest prevalence period for the parameter of interest was not appropriate (e.g. lifetime prevalence) | The prevalence period is the period that the subject is asked about e.g. “Have you experienced low back pain over the previous year?” In this example, the prevalence period is one year. The longer the prevalence period, the greater the likelihood of the subject forgetting if they experienced the symptom of interest (e.g. low back pain). Examples:  Subjects were asked about pain over the past week. The answer is: Yes (LOW RISK).  Subjects were only asked about pain over the past three years. The answer is: No (HIGH RISK). |
| 10. Were the numerator(s) and denominator(s) for the parameter of interest appropriate? | Yes (LOW RISK): The paper presented appropriate numerator(s) AND denominator(s) for the parameter of interest (e.g. the prevalence of low back pain).  No (HIGH RISK): The paper did present numerator(s) AND denominator(s) for the parameter of interest but one or more of these were inappropriate. | There may be errors in the calculation and/or reporting of the numerator and/or denominator. Examples:  There were no errors in the reporting of the numerator(s) AND denominator(s) for the prevalence of low back pain. The answer is: Yes (LOW RISK).  In reporting the overall prevalence of low back pain (in both men and women), the authors accidentally used the population of women as the denominator rather than the combined population. The answer is: No (HIGH RISK). |

* **Items in original Hoy tool that are excluded:**

items 2-3: there is no known sampling frame for sexual or gender minorities; therefore, assessing this is not possible; nor is random selection possible

item 7: there are no measures of SOGIECE or CT that have been tested for psychometric properties
