# Supplementary material for: The scope and nature of sexual orientation and gender identity and expression change efforts: a systematic review protocol
Source: Syst Rev. 2021 Jan 8;10:14. doi: 10.1186/s13643-020-01563-8 (PMC7796537; doi:10.1186/s13643-020-01563-8)
Supplement: Supplementary file 2 — Additional file 2. Medline search strategy was peer-reviewed using the Peer Review of Electronic Search Strategies (PRESS) checklist [file 13643_2020_1563_MOESM2_ESM.docx]

**Appendix B. Medline search strategy was peer-reviewed using the Peer Review of Electronic Search Strategies (PRESS) checklist**

Database: Ovid MEDLINE(R) <1946 to May 29, 2020> Search Strategy:

--------------------------------------------------------------------------------

1     "conversion therap*".mp.

2     "conversion effort*".mp.

3     "conversion practice*".mp.

4     or/1-3

5     "“reparative therap*".mp.

6     "“reparative effort*".mp.

7     "“reparative practice*".mp.

8     or/5-7

9     "reorientation therap*".mp.

10     "sexual reorientation".mp.

11     "gender reorientation".mp.

12     "gender identity reorientation".mp.

13     or/9-12

14     "sexual orientation change*".mp.

15     "gender identity change*".mp.

16     "gender expression change*".mp.

17     or/14-16

18     "ex-gay".mp.

19     "gender acceptance therap*".mp.

20     "reintegrative therap*".mp.

21     "gay cure therap*".mp.

22     "sexual identity fluidity exploration".mp.

23     "psychological attempts to change a person’s gender identity from transgender to cisgender".mp.

24     PACGI.mp.

25     or/18-24

26     4 or 8 or 13 or 17 or 25

27     Bisexuality/

28     exp homosexuality/

29     exp "Sexual and Gender Minorities"/

30     bisexual*.mp.

31     homosexual*.mp.

32     "men who have sex with men".mp.

33     sexual orient*.mp.

34     "women who have sex with women".mp.

35     "sexual minorit*".mp.

36     (gay* or lesbian*).mp.

37     (GLB* or LGB*).mp.

38     (queer* or two spirit*).mp.

39     (nonheterosexual* or non-heterosexual*).mp.

40     Transgender Persons/

41     transgender*.mp.

42     "gender divers*".mp.

43     "gender creativ*".mp.

44     (non-binary and gender).mp.

45     genderqueer*.mp.

46     genderfluid.mp.

47     "trans wom*".mp.

48     "trans m*".mp.

49     "transwom*".mp.

50     "gender affirm*".mp.

51     (mtf or ftm).mp.

52     (transfeminine or transmasculine).mp.

53     "sex* reassignment”".mp.

54     ("gender identity disorder*" or GID).mp.

55     (transex* or transsex* or trans sex*).mp.

56     Transsexualism/

57     "gender dysphori*".mp.

58     or/27-57

59     26 and 58
